# Dual effects of thyroid hormone on neurons and neurogenesis in traumatic brain injury

**DOI:** 10.1038/s41419-020-02836-9

**Published:** 2020-08-09

**Authors:** Chao Lin, Nan Li, Hanxiao Chang, Yuqi shen, Zheng Li, Wu wei, Hua Chen, Hua Lu, Jing Ji, Ning Liu

**Affiliations:** 1 Department of Neurosurgery, the First Affiliated Hospital of Nanjing Medical University, Nanjing 210029, China; 2 Department of Neurosurgery, Jiangsu Province Hospital, Nanjing 210029, China; 3 Department of Nephrology, Drum Tower Hospital, Nanjing 210029, China

**Keywords:** Cell death in the nervous system, Neural stem cells

## Abstract

Thyroid hormone (TH) plays a crucial role in neurodevelopment, but its function and specific mechanisms remain unclear after traumatic brain injury (TBI). Here we found that treatment with triiodothyronine (T3) ameliorated the progression of neurological deficits in mice subjected to TBI. The data showed that T3 reduced neural death and promoted the elimination of damaged mitochondria via mitophagy. However, T3 did not prevent TBI-induced cell death in phosphatase and tensin homolog (PTEN)-induced putative kinase 1 (*Pink1*) knockout mice suggesting the involvement of mitophagy. Moreover, we also found that T3 promoted neurogenesis via crosstalk between mature neurons and neural stem cells (NSCs) after TBI. In neuron cultures undergoing oxygen and glucose deprivation (OGD), conditioned neuron culture medium collected after T3 treatment enhanced the in vitro differentiation of NSCs into mature neurons, a process in which mitophagy was required. Taken together, these data suggested that T3 treatment could provide a therapeutic approach for TBI by preventing neuronal death via mitophagy and promoting neurogenesis via neuron–NSC crosstalk.

## Introduction

Traumatic brain injury (TBI) is considered to be a leading cause of substantial mortality and long-term disability among young adults worldwide^1^. In the United States, TBI contributes to >30% of the deaths resulting from traumatic injury each year, with ~1.4 million cases requiring emergency treatment and 235,000 being hospitalized^2,3^. An estimated 85,000 TBI survivors per year suffer from long-term complications, including cognitive disorders, chronic disability, and persistent vegetative states^2,4^. The economic burden is serious; about $37.8 billion is spent annually on treatments, long-term care, work absences, and premature deaths^2,5^. However, no specific therapies have been developed, while clinical treatments focus only on preventing complications or providing support in nature.

Thyroid hormone (TH) is crucial for neural stem cell (NSC) differentiation and brain development; its deficiency leads to irreversible neurological alterations^6,7^. TBI is well known to cause changes in systemic TH levels^8^. Accumulating evidence from clinical and experimental studies has indicated that TH treatment showed neuroprotective properties after acute brain injury, including stroke and TBI^9–11^. Understanding TH’s actions and the mechanisms regulating TH functions in the adult brain is essential to design potential therapeutic strategies for TBI, due to dysregulated TH signaling during this procession.

Mitochondria are indispensable organelles for energy production, cell signaling, and cell survival in eukaryotic cells. However, damaged and dysfunctional mitochondria are also responsible for generating the majority of reactive oxygen species (ROS) and the release of cytochrome *c* (cyt *c*), which results in cell death^12^. Thus selective elimination of damaged mitochondria by mitophagy could be a potential therapeutic target for TBI. Although several studies have shown that TH coordinately regulates mitophagy and mitochondrial function in the lungs and muscle in a coordinated fashion, none have done so in a TBI model^13,14^.

In this study, triiodothyronine (T3)-mediated restoration of TBI-induced mitochondrial dysfunction was associated with the inhibition of mitochondria-regulated neuronal apoptosis that depended on phosphatase and tensin homolog (PTEN)-induced putative kinase 1 (Pink1)-mediated mitophagy. Furthermore, we found that T3 indirectly regulated neurogenesis by modifying neuron–NSC crosstalk. Taken together, these findings suggested that TH treatment could reduce neuronal death, promote NSCs to mature neurons, and subsequently ameliorate behavioral deficits after TBI.

## Methods

### Mice and protocol

This study used male C57BL/6 mice (12 weeks old) weighing 22–26 g. We obtained *Pink1*^−/−^ mice from the Model Animal Resource Center (MARC; Nanjing, China)^15^. *Pink1* knockout resulted in the impairment of mitophagy. These mice did not present other specific abnormalities at baseline. Green fluorescent protein (GFP) microtubule-associated protein light chain 3 (LC3) transgenic mice were obtained from CasGene Biotech. Co., Ltd (Beijing, China)^16^. We crossed GFP-LC3 mice and *Pink1*^−/−^ mice to generate GFP-LC3 *Pink1*^−/−^ mice. All transgenic mice were of C57BL/6J background. Standard laboratory animal food and water were provided by the Animal Center of Nanjing Medical University (NMU; Nanjing China). Mice were maintained in a pathogen-free environment and housed on 12-h day/night cycles at an ambient temperature of 22 ± 2 °C. All animal experiments were approved by the Animal Care and Use Committee of NMU.

Cortical lesion volume and brain water content were measured on day 3 after brain injury. Behavioral tests were done on post-TBI days 7–13. On day 14 after brain injury, mice were sacrificed and samples were obtained for other designed experiments.

### Drug treatment

We subcutaneously administered T3 (catalog no. T2877; Sigma-Aldrich, St. Louis, MO, US) to mice at 20 μg/100 g/day for 14 consecutive days, starting 24 h after brain injury. Saline (vehicle) was used in the sham group. In vitro, cells were treated with 10 nM T3 for 72 h.

### Traumatic brain injury

Controlled cortical impact was applied to induce cerebral contusion as previously described^16^. Briefly, a craniotomy was done with a portable drill and 4-mm trephine over the right parieto-temporal cortex. The bone flap was removed and the dura mater was left intact. Injury was delivered with a 3-mm flat-tip impounder at a velocity of 6 m/s, a depth of 0.6 mm, and a duration of 100 ms. The bone flap was discarded and the scalp was sutured and closed. Then the mice were returned to their cages to recover from anesthesia. Similarly, mice without impact were considered as sham.

### Cortical lesion volume

One section was obtained every 0.5 mm for lesion volume measurement. Lesion volume was calculated from the summation of areas of defect on each slice with Image J (National Institutes of Health, Bethesda, MD, USA) and multiplied by slice thickness.

### Brain water content

The mouse brain was immediately removed and weighed after sacrifice to obtain the wet weight. Then it was dried at 70 °C for 72 h to obtain the dry weight. Lastly, the brain water content was measured in accordance to water content (%) = [(wet weight − dry weight)/wet weight] × 100%.

### Cell culture

Primary neurons were isolated from 17-day-old embryonic mouse brain cerebral cortex and plated on poly-d-lysine-coated culture dishes (2.0 × 10^6^ cells/mL). In accordance with this study protocol, we used primary neurons for experiments at 7 days. NSCs were isolated from 14-day-old embryonic mouse brains (the lateral ganglionic eminence and the ventral midbrain of embryonic) and plated on laminin- and poly-l-ornithine-coated dishes. In accordance with the study protocol, NSCs were grown to 70% confluence before designed experiments.

### Oxygen–glucose deprivation (OGD)

In brief, the medium was changed to Dulbecco’s modified Eagle’s medium (DMEM) without glucose and serum. Hypoxia was induced by placing the cells in a hypoxic chamber (Billups–Rothenberg, Inc., San Diego, CA, US), which we perfused by 90% N_2_, 5% H_2_, and 5% CO_2_ for 30 min to obtain <0.5% O_2_. Then the chamber was sealed and kept at 37 °C. Primary neurons were incubated at 37 °C for 2 h before reperfusion, while for NSCs, OGD duration was 8 h.

### Conditioned medium (CM) preparation

Primary neurons were incubated in DMEM without serum for 2 h, with or without OGD, and then treated with 10 nM T3 for 24 h. Next, the conditioned medium (referred as OGD-T3-N-CM or control-T3-N-CM) was collected. In accordance with the experimental protocols, NSCs with or without OGD were treated with CM and normal medium at a ratio of 1:1 at 37 °C for 24 h.

### Oxygen consumption rate (OCR)

We assessed the OCR of primary neurons using a microplate (type XF24) extracellular analyzer (Seahorse Bioscience, Billerica, MA, US). Briefly, we added 1 μM oligomycin to inhibit adenosine triphosphate synthesis. Then 1 μM carbonyl cyanide-4-(trifluoromethoxy) phenylhydrazone (FCCP) was added to uncouple the mitochondrial membrane. Finally, we added 1 μM of each rotenone and antimycin A (R+A) to inhibit complexes I and III. Basal OCR was computed as [OCR_initial_ − OCR_R+A_]; maximum respiration rate was measured as [OCR_FCCP_ − OCR_R+A_].

### Confocal microscopy

Briefly, coronal frozen sections were embedded onto the slides and then incubated by primary or secondary antibodies. Multiple fields from one coverslip/well were selected to get an average at random and imaged for further analysis using the Image-Pro Plus 7.0 software. Each experiment was repeated independently in triplicate or more. GFP-LC3 mice were used in this experiment. The following primary antibody was used: cyclooxygenase-4 (COXIV; 1:1000; Abcam, catalog no. ab16056).

### Terminal deoxynucleotidyl transferase dUTP nick-end labeling (TUNEL) assay

TUNEL assay was used to evaluate apoptotic cell death in accordance with the manufacturer’s instruction (#12156792910, Roche, Germany). Briefly, coronal frozen sections (7-μm thick) were embedded onto the slides and incubated with 50 μL TUNEL reaction mixture. The slides were observed and quantified using a Nikon fluorescent microscope. The apoptosis was calculated as TUNEL-positive cells (red)/4,6-diamidino-2-phenylindole (DAPI) (blue).

### Immunoblot analysis

Extracts for western blot were homogenized in RIPA buffer. In brief, proteins were separated by electrophoresis and then transferred to polyvinylidene difluoride membranes, which were processed with primary antibodies. Next, we incubated these membranes in the appropriate diluted secondary antibody at room temperature for 1 h. The follow primary antibodies were used: p62 (1:1000; Abcam, catalog no. ab56416), LC3 (1:1000; Cell Signaling Technology, catalog no. 2775s), translocase of outer mitochondrial membrane 40 homolog (TOM40; 1:1000; Abcam, catalog no. ab51884), COXIV (1:1000; Cell Signaling Technology, catalog no. 11967), manganese superoxide dismutase (MnSOD; 1:1000; Cell Signaling Technology, catalog no. 13141), β-actin (1:1000; Cell Signaling Technology, catalog no. 3700), and NeuN (1:1000; Abcam, catalog no. ab104224).

### Transmission electron microscopy

For microscopic examination, mice were euthanized with 10% choral hydrate administration and perfused using 4% paraformaldehyde. The ultrastructure of the samples (100-nm ultrathin sections) was observed with a transmission electron microscope (Quanta 10, FEI Co.).

### Determination of ROS

We determined ROS in brain tissues using dihydroethidium (DHE; catalog no. D11347; Thermo Fisher) staining. On day 14 after brain injury, mice were sacrificed and samples were obtained for DHE experiment. Briefly, we obtained 20-μm-thick cryosections of frozen brain tissues. The samples were incubated with 10 μM DHE at 37 °C for 30 min in a humidified, dark chamber and then counterstained with DAPI. Fluorescent images were obtained using the Zeiss confocal microscope and determined with Image J.

### Morris water maze (MWM)

MWM was performed by the same experimenter to evaluate spatial learning and memory at the same time each day between 08:30 and 12:00 hours. For each trial, mice underwent this experiment using a random start locations. If mice failed to reach the platform within setting time, they were picked up and placed on the platform for 15 s. For probe trials, mice were placed in the tank opposite the target quadrant and looked for the platform for 60 s. The hidden platform trial was done on test days 1–5. On test day 5, a probe trial was performed 2 h after the hidden platform trial. Both visible platform trials were done on test day 6 or 7. The latencies to find the platform and the number of entries were analyzed with a tracking device and software (Chromotrack 3.0, San Diego Instruments).

### Elevated plus maze

The elevated plus maze was done to evaluate anxiety/risk-taking behavior (Noldus Ethovision). The elevated plus maze is raised 85 cm above the floor and comprised of the open center (decision zone), the two open arms (aversive arms), and the two closed arms (safe arms). These four arms were extended out opposite from each other to create a plus shape. Mice were placed on the center platform, facing a closed arm, and allowed to explore the maze for 5 min. Time in the open arms was used to quantify anxiety-like behavior.

### Novel object recognition (NOR)

Briefly, mice were placed on an empty surface for 5 min on test day 1. On test day 2, mice were placed on the same surface including two identical objects for 5 min to recognize them. Then mice were placed on this surface including two objects, one is “old” object and the other is “new” object, for 5 min on test day 3. The amount of time spent in exploring “new” object was recorded to evaluate the extent of learning and memory.

### Neurological severity score (NSS)

In brief, a ten-point NSS was used to assess motor function and reflexes of the mice at different time points: (i) exit circle: ability to exit circle of 30 cm diameter (time limit: 3 min); (ii) hemiparesis: paresis of upper and/or lower limb of the contralateral side; (iii) straight walk: ability to walk straight on the floor; (iv) startle reflex: innate reflex; the mouse will bounce in response to a loud hand clap; (v) seeking behavior: physiological behavior as a sign of “interest” in the environment; (vi) beam balancing: ability to balance on a beam of 7 mm width for at least 10 s; (vii) grip on a round stick: ability to grip on a beam of 5 mm width for at least 10 s; (viii) beam walk: ability to cross a 30-cm long beam of 3 cm width; (ix) beam walk: same task, on a 2-cm wide beam; and (x) beam walk: same task, on a 1-cm wide beam. One point is given for failing to complete each of the tasks.

### Statistical analysis

All results are reported as the mean ± standard deviation (SD). Gray levels were detected with Image J. The unpaired Student’s *t* test was applied to compare the differences between two groups with the Prism Software 6.04 (GraphPad Software, Inc.). Motor and MWM data were analyzed by two-way analysis of variance for an overall statistic and followed by Tukey’s test for between-group comparisons. Significant differences were defined as *P* value < 0.05.

## Results

### T3 treatment rescued behavioral deficits after TBI

**Fig. 1:**
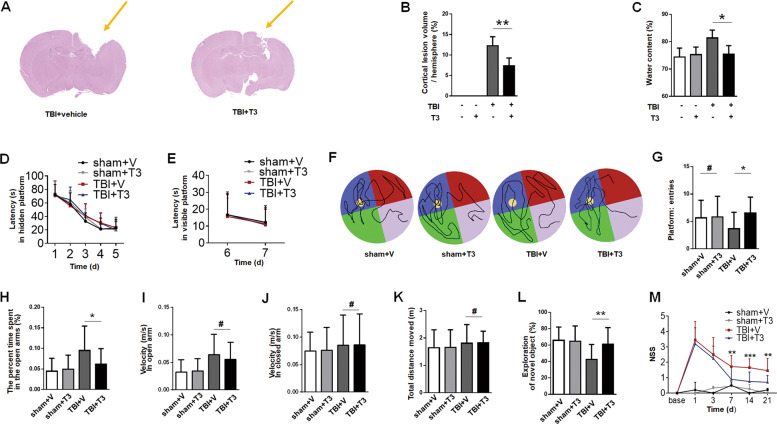
T3 treatment rescued histological and functional deficits after TBI. **a**, **b** Representative images (**a**, arrow) and quantification (**b**) of cortical lesion volume (*n* = 5, **P < 0.01). **c** Analysis of water content (*n* = 5, *P < 0.05). **d**–**g** Morris water maze (MWM). Graph showing hidden platform trial (**d**) and visible platform trials (**e**). Representative images (**f**) and quantification of probe tests (**g**, ^#^*P* > 0.05, *P < 0.05). **h**–**k** Elevated plus maze. In the open arm, T3-treated TBI mice had similar performance to sham in the present time (**h**), but vehicle-treated TBI mice spent more time (**h**, *P < 0.05). **i** Velocity in the open arm (^#^*P* > 0.05). **j**, **k** All groups had similar velocity in the closed arm (**j**, ^#^*P* > 0.05) and traveled similar total distance (**k**, ^#^*P* > 0.05). **l** Novel object recognition (NOR, **P < 0.01). **m** Neurological severity score (NSS, **P < 0.01, ***P < 0.001 between T3- and vehicle-treated TBI mice). Sham mice, *n* = 20; T3-treated sham mice, *n* = 20; vehicle-treated TBI mice, *n* = 20; T3-treated TBI mice, *n* = 22. All data are mean ± SD. V vehicle.

To evaluate the effect of T3 on outcome after TBI, we examined lesion volume and brain edema. First, we found that cortical lesion volume was smaller in the T3-treated mice than the mice that received vehicle (Fig. 1a, b). Furthermore, the brain water content was significantly decreased in the TBI group treated with T3 versus those treated by vehicle (Fig. 1c), suggesting that T3 had the effect of ameliorating brain edema induced by TBI. Taken together, these data demonstrated that T3 treatment diminished histological defects after TBI.

To further determine the impact of T3 on behavioral or functional outcomes after TBI, we assessed the mice using MWM, elevated plus maze, NOR, and NSS after injury. Mice from different groups showed no differences in the hidden- or visible-platform trial performance (Fig. 1d–g). However, we observed a significant improvement during the probe test in the TBI + T3 group compared with the TBI + vehicle group (Fig. 1f, g). Latency in visible-platform trials did not differ among groups, indicating that MWM performance was not influenced by differences in swimming ability or vision (Fig. 1e). During the elevated plus maze test, T3-treated mice exhibited minimal risk-taking performance, similar to sham mice (Fig. 1h–k). We found that TBI mice treated with T3 spent less time in the open arm than vehicle-treated TBI mice (Fig. 1h). All groups had similar velocity in the open (Fig. 1i) and closed arms (Fig. 1j). Nest, we conducted an NOR test to further evaluate memory retention. T3-treated mice spent significantly more time in exploring the novel object than the vehicle-treated mice (Fig. 1l). There was a tendency for T3 treatment to normalize the performance of mice on these tests. Finally, mice treated with T3 showed improved motor skills (Fig. 1m). Taken together, these data suggested that T3 administration resulted in a possible reduction of behavioral deficits and restored some functional outcomes after TBI.

### T3 treatment promoted autophagy after TBI

**Fig. 2:**
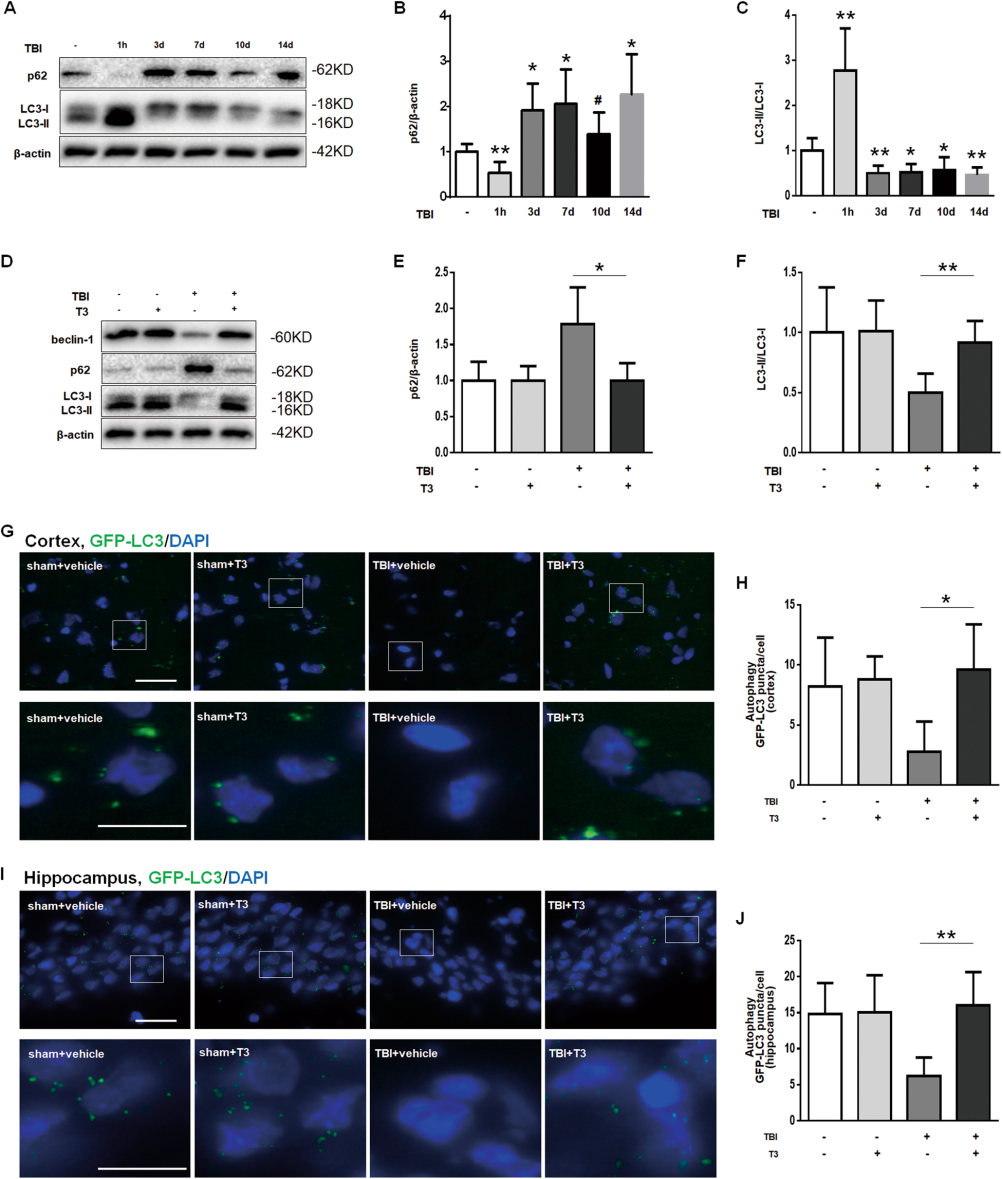
T3 treatment promoted mitophagy after TBI. **a**–**c** Western blot analysis of autophagy after TBI. β-Actin was used as a loading control (*P < 0.05, **P < 0.01, ^#^*P* > 0.05 versus sham). **d**–**f** T3 restored the activity of autophagy after TBI (*P < 0.05, **P < 0.01). In **a**–**f**, β-actin was used as loading control and protein was obtained from cortical injured lesion. **g** Representative fluorescence micrographs of mouse cortex. Scale bar: up 50 µm, down 20 µm. **h** Quantifications of LC3 puncta in the mouse cortex, as an indicator of autophagosome (*P < 0.05). **i** Representative fluorescence micrographs of the mouse hippocampus. Scale bar: up 50 µm, down 20 µm. **j** Quantifications of LC3 puncta in the mouse hippocampus, as an indicator of autophagosome (**P < 0.01).

We have recently shown that autophagy can attenuate neuronal death and behavioral deficits after TBI^12,16^. As other studies have reported, T3 induces autophagy in brown adipose tissue and lungs^13,17^. We wondered whether T3 plays an important role in autophagy after TBI. Western blot analysis showed an increase in p62 (SQSMT1) and a decrease in lipidated microtubule-associated protein LC3–phosphatidylethanolamine conjugate (LC3-II) in TBI versus control brain tissue, suggesting inhibition of autophagy at least from 3 to 14 days after head injury (Fig. 2a–c). Strikingly, we found that T3 treatment restored the autophagic activity as evidenced by decreased p62 and increased LC3-II (Fig. 2d–f). Furthermore, representative immunofluorescence data showed that T3 treatment promoted the expression of GFP-LC3 in the cortex (Fig. 2g, h) and hippocampus (Fig. 2i, j), suggesting an increase in autophagy. Taken together, these data showed that T3 treatment could restore the autophagic activity and promote the brain autophagy after TBI.

### T3-promoted mitophagy was dependent on the *Pink1* pathway

**Fig. 3:**
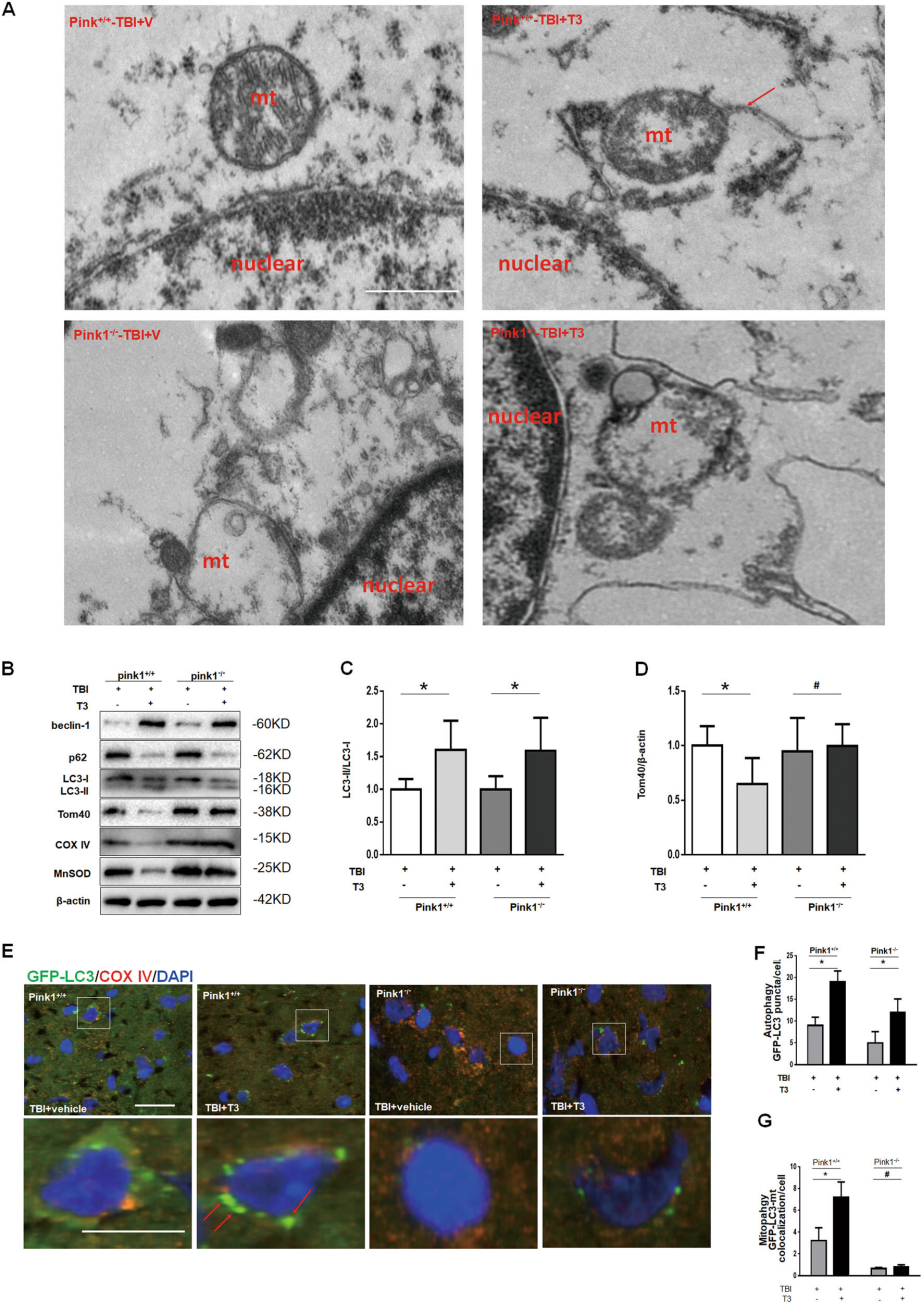
T3 treatment promoted mitophagy via Pink1 pathway. **a** Representative electron micrographs of the mouse cortex. Mitochondrion was engulfed by vacuolar structures (arrow). Mt mitochondrion. Scale bar, 1 µm. **b**–**d** T3 decreased of the mitochondrial markers of TOM40, MnSOD, and COXIV in *Pink1*^*+/+*^ mice but not in *Pink1*^−/−^ mice (*P < 0.05, ^#^*P* > 0.05). **e**–**g** T3 increased co-localization of GFP-LC3 and mitochondria (arrows) versus control in *Pink1*^*+/+*^ mice after TBI but not in *Pink1*^−/−^ mice (Scale bar, 20 µm. *P < 0.05, ^#^*P* > 0.05).

As previously reported, T3 improved mitochondrial bioenergetics and ameliorated mitochondrial dysfunction via mitophagy^13,17^. *Pink1* is a positive regulator of mitophagy; therefore, to confirm the role of mitophagy, we subjected *Pink1*^*+/+*^ or *Pink1*^−/−^ mice to TBI^18^. Compared with controls, we observed an increased amount of autophagic vacuoles containing damaged mitochondria in the *Pink1*^*+/+*^ mice but not in the *Pink1*^−/−^ mice (Fig. 3a). Next, we found that T3-induced decreases in mitochondrial marker proteins from inner mitochondrial membrane (COXIV), outer mitochondrial membrane (TOM40), and matrix (MnSOD) subcompartments were partially reversed by genetic deletion of *Pink1* (Fig. 3b–d). Using GFP-LC3 transgenic mice, we found that T3 treatment enhanced co-localization of GFP-LC3-positive puncta with mitochondria and decreased the expression of mitochondrial markers, which suggested an increase in mitophagy (Fig. 3e–g). Furthermore, we found that T3-induced mitophagy was blocked by genetic deletion of *Pink1* (Fig. 3e, g). Taken together, these data showed that T3 treatment significantly promoted mitophagy after TBI dependent on the *Pink1* pathway.

### T3 treatment reversed TBI-induced mitochondrial dysfunction via mitophagy

**Fig. 4:**
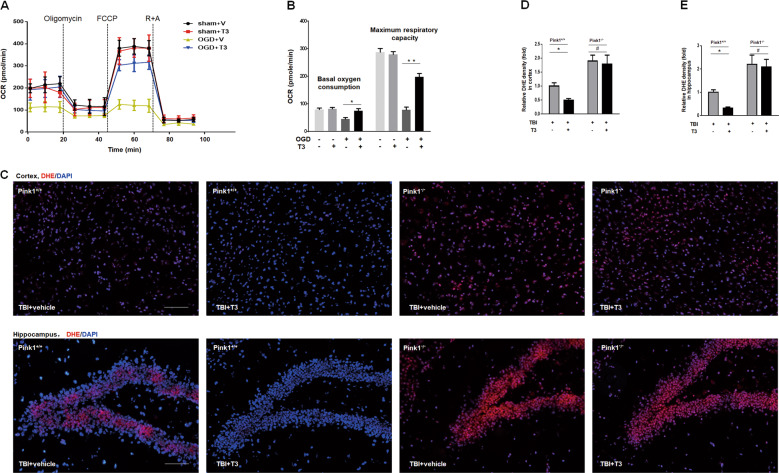
T3 treatment attenuated mitochondrial dysfunction after TBI. **a** Seahorse analysis of OCR for neurons subjected to hypoxia and then treated with or without 10 nM T3 for 24 h. **b** A graph showing basal and maximal OCR (*P < 0.05, **P < 0.01). **c** Representative images of DHE staining on the cortex (up) and hippocampus (down), Scale bar, 100 µm. **d** Quantifications of DHE fluorescence intensity on the cortex (*P < 0.05, ^#^*P* > 0.05). **e** Quantifications of DHE fluorescence intensity on the hippocampus (*P < 0.05, ^#^*P* > 0.05).

Given the role of T3 in promoting mitophagy, we examined its effect on mitochondrial dysfunction caused by TBI. One pathway that neurons employ to prevent apoptosis is via effective elimination of damaged mitochondria, thus preventing excessive ROS production through mitophagy. First, we found that T3 treatment increased basal OCR and maximum respiratory capacity in primary neurons with OGD (Fig. 4a, b). Next, we assessed ROS levels in the cortex or hippocampus using DHE staining, which is commonly applied to detect ROS production. As expected, we observed DHE signal in the cortex (Fig. 4c up, Fig. 4d) and hippocampus (Fig. 4c down, Fig. 4e) after TBI, and it was significantly attenuated by T3 treatment. However, ROS level in *Pink1*^−/−^ mice was not reduced by such treatment (Fig. 4c–e). Taken together, these data showed that T3 treatment could ameliorate mitochondrial dysfunction induced by TBI in a manner that depended on the mitophagy pathway.

### T3 treatment suppressed mitochondria-regulated apoptosis in a mitophagy-dependent manner

**Fig. 5:**
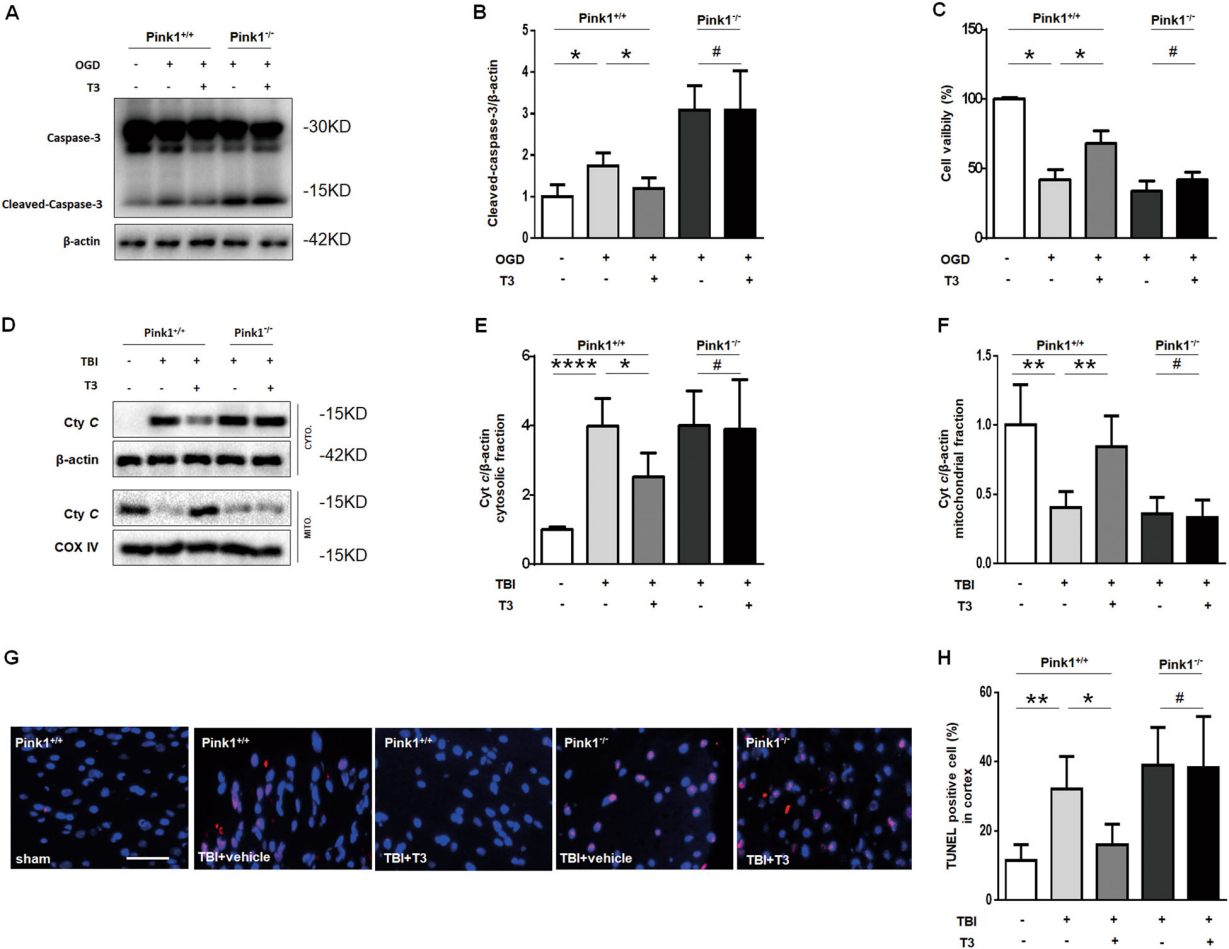
T3 treatment reduced cell death after TBI. **a** Representative western blot analysis for the expression of caspase-3 and cleaved-caspase-3 in cortical neuronal cultures that were treated as indicated. β-Actin was used as a loading control. **b** Densitometry of cleaved-caspase-3 (*P < 0.05, ^#^*P* > 0.05). **c** Pericyte viability, as determined by the Cell Counting Kit (CCK)-8, in cortical neuronal cultures that were treated as indicated (*P < 0.05, ^#^*P* > 0.05). **d** Representative western blot analysis of cytochrome *c* (cyt *c*) in the injured cortex. **e** Densitometry of cyt *c* in cytosolic fraction (*P < 0.05, ****P < 0.0001, ^#^*P* > 0.05). **f** Densitometry of cyt *c* in mitochondrial fraction (**P < 0.01, ^#^*P* > 0.05). **g**, **h** Representative fluorescence micrographs of the mouse cortex after TBI. Apoptotic cell death was assessed by TUNEL staining (scale bar, 50 µm. *P < 0.05, **P < 0.01, ^#^*P* > 0.05). In **d**–**h**, samples were obtained from the cortical injured lesion on day 14 after brain injury.

Given the ability of T3 to restore mitochondrial function, we asked whether it could prevent TBI-induced neuronal death. OGD of primary cortical neuron cultures in vitro upregulated the level of cleaved-caspase-3 and caused cytotoxicity (Fig. 5a, b). The addition of T3 at the onset of OGD significantly blocked induction of this pathway (Fig. 5a, b) and rescued neuron viability (Fig. 5c). However, we did not find this change in primary cortical neuron from *Pink1*^−/−^ mice (Fig. 5a–c).

To confirm this effect of T3, we subjected *Pink1*^*+/+*^ or *Pink1*^−/−^ mice to TBI. Cyt *c* released into the cytosol is an apoptogenic factor that triggers neuronal death^12^. Our previous study showed that the elimination of damaged mitochondria reduced the release of cyt *c* and subsequently prevented mitochondria-dependent apoptosis after TBI^12^. As expected, T3 could significantly inhibit cyt *c* release in *Pink1*^*+/+*^ mice subjected to TBI (Fig. 5d–f). Consistent with the data in vitro, we did not observe this inhibitory activity of T3 on TBI-induced cell death in *Pink1*^−/−^ mice, suggesting a central role for mitophagy in this process (Fig. 5d–f). Compared with mice treated by vehicle, T3 treatment reduced the number of TUNEL-positive cells in *Pink1*^*+/+*^ mice after TBI but not in *Pink1*^−/−^ mice (Fig. 5g, h). In addition, we found that *Pink1* deficiency significantly increased the death of dopaminergic neurons in the ventral mesencephalon after TBI but did not cause more neuronal death in the injured cortex (Fig. 5 and Supplementary Fig. S1). Taken together, these data showed that T3 treatment could significantly prevent TBI-induced neuronal death via clearance of damaged mitochondria by mitophagy*.*

### T3 treatment enhanced neurogenesis after TBI

**Fig. 6:**
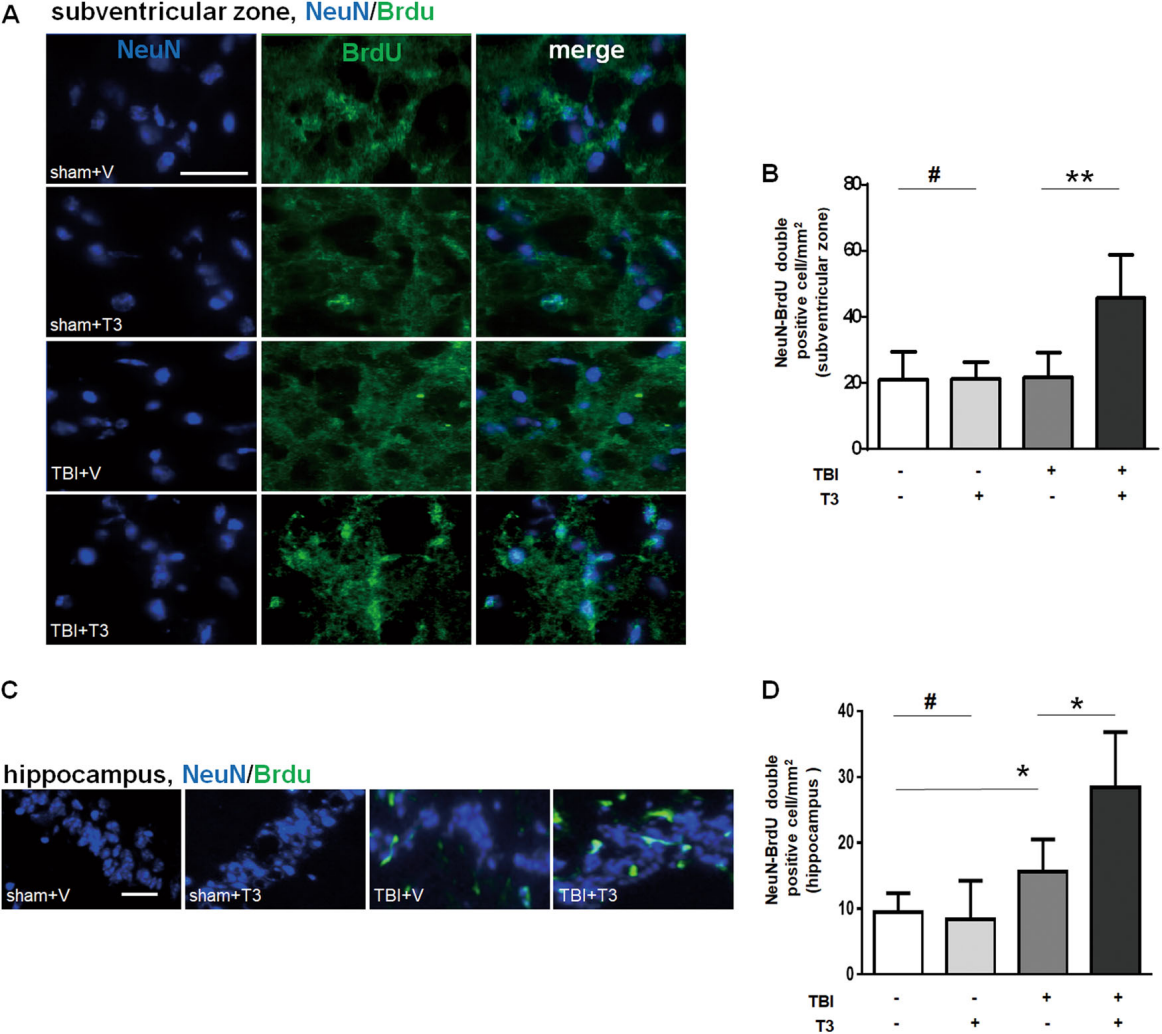
T3 treatment promoted neurogenesis after TBI. **a** Representative images of BrdU^+^NeuN^+^ double-positive cells in subventricular zone (scale bar, 50 µm). **b** Quantification of double-positive cells in subventricular zone (**P < 0.01, ^#^*P* > 0.05). **c** Representative images of BrdU^+^NeuN^+^ double-positive cells in the hippocampus (scale bar, 50 µm). **d** Quantification of double-positive cells in the hippocampus (*P < 0.05, ^#^*P* > 0.05).

TBI is well known to induce delayed processes of endogenous neurogenesis^19^. We asked whether T3 could exert these additional beneficial effects by stimulating neurogenesis. We found that T3 treatment seemed to increase the number of cells that were positive for both NueN and 5-bromo-2′-deoxyuridine (BrdU) in the subventricular zone (Fig. 6a). Quantification of the NeuN^+^/BrdU^+^ cells showed that T3 treatment significantly increased these surrogate markers of neurogenesis, compared with that in vehicle-treated mice that were subjected to TBI (Fig. 6b). Similarly, we observed more NeuN^+^/BrdU^+^ cells in hippocampus of mice treated by T3 than those treated by vehicle (Fig. 6c, d). In addition, we also found that T3 treatment could promote neurogenesis on day 3 after brain injury (Supplementary Fig. S2). Taken together, these data showed the effect of T3 on adult neurogenesis in our model.

### T3 treatment promoted neurogenesis via the crosstalk between mature neurons and NSCs in our model

**Fig. 7:**
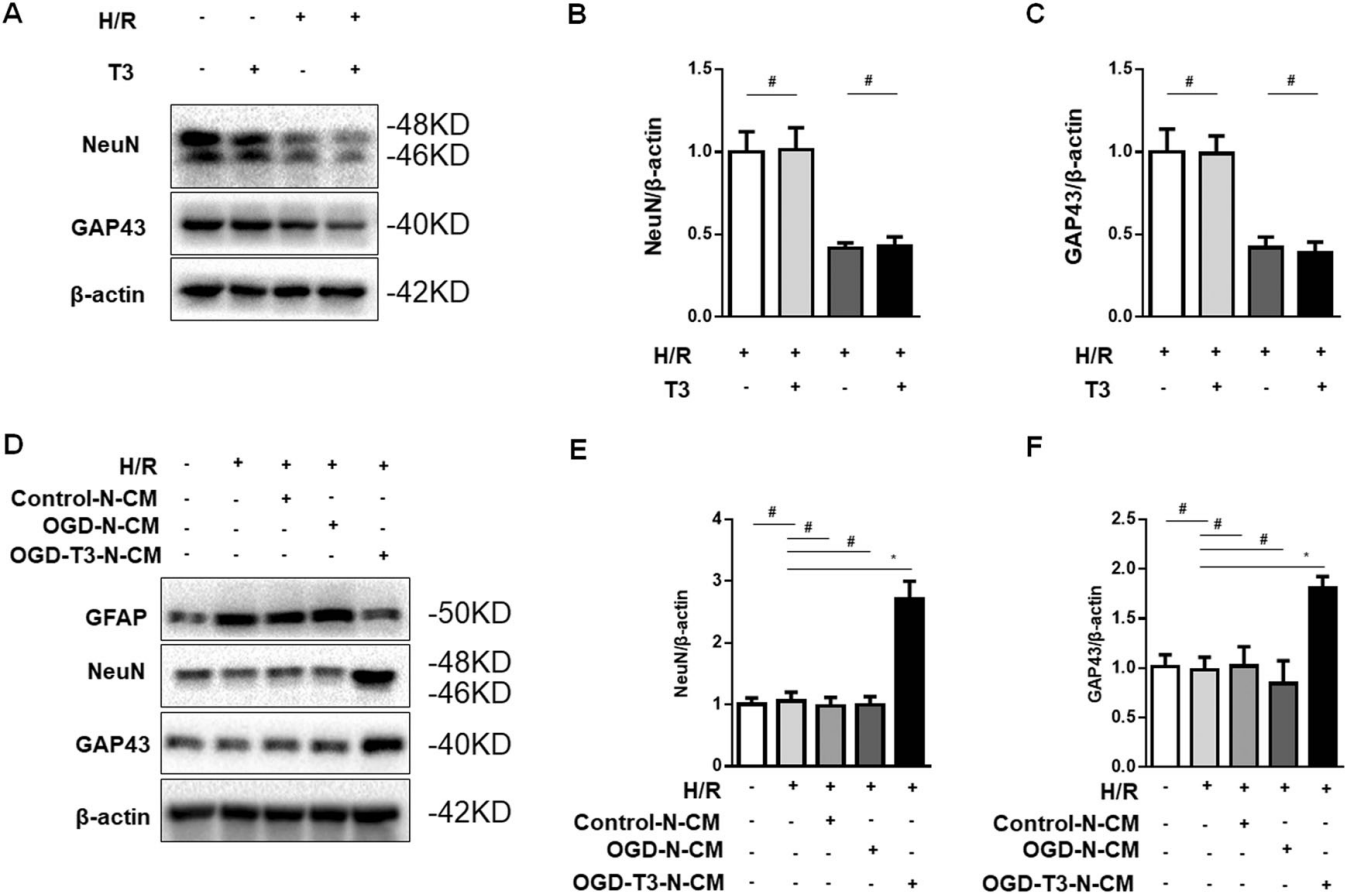
T3 treatment promoted neurogenesis via crosstalk between mature neuron and NSCs. **a**–**c** Representative western blot and quantification of NeuN (**b**, ^#^*P* > 0.05) and growth-associated protein 43 (GAP43; **c**, ^#^*P* > 0.05) expression in NSC cultures that were treated with T3 or vehicle for 3 days in a reoxygenation state after 8 h of hypoxia (H/R). **d**–**f** Representative western blot and quantification of the indicated proteins in NSC cultures that were subjected to H/R and treated with mature neuron conditioned medium from the experimental treatment groups. **e** Quantification of NEUN (*P < 0.05, ^#^*P* > 0.05). **f** Quantification of GAP43 (*P < 0.05, ^#^*P* > 0.05).

To explore the specific molecular mechanism of neurogenesis, we first subjected cultured primary mouse NSCs to hypoxia–reoxygenation. T3 treatment could not promote differentiation of these NSCs to mature neurons (Fig. 7a–c). Next, we wanted to know whether mature neurons could promote NSC differentiation. The addition of neuron-conditioned medium (with or without OGD) could not shift NSCs toward a neuronal phenotype (Fig. 7d–f). However, conditioned medium from cultured neurons that were subjected to OGD and treated with T3 enhanced the differentiation of NSCs into mature neurons (Fig. 7d–f). Taken together, these data showed that T3 treatment promoted neurogenesis via crosstalk between mature neurons and NSCs.

### The *Pink1* pathway was required for T3-promoted neurogenesis in our model

**Fig. 8:**
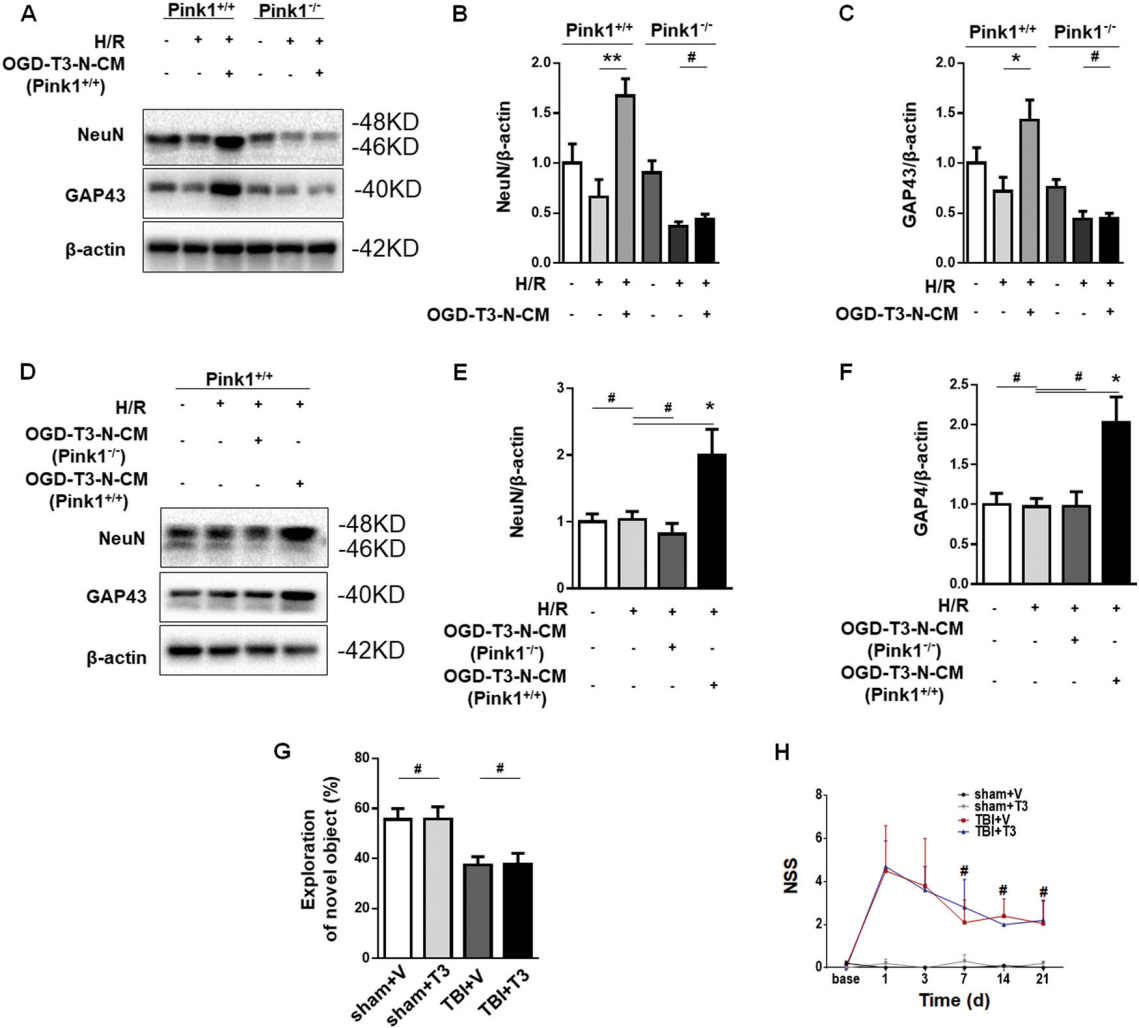
Mitophagy was essential for T3-mediated neurogenesis. **a**–**c** Representative western blot and quantification of NeuN and GAP43 in NSC cultures isolated from *Pink1*^*+/+*^ or *Pink1*^−/−^ mice. **b** Quantification of NeuN (**P < 0.01, ^#^*P* > 0.05). **c** Quantification of GAP43 (*P < 0.05, ^#^*P* > 0.05). **d**–**f** Representative western blot analysis of NeuN and GAP43 in NSC cultures treated by different conditioned mediums. **e** Quantification of NeuN (*P < 0.05, ^#^*P* > 0.05). **f** Quantification of GAP43 (**P < 0.01, ^#^*P* > 0.05). **g** Novel object recognition (NOR, ^#^*P* > 0.05). **h** Neurological severity score (NSS, ^#^*P* > 0.05 versus vehicle-treated TBI mice). Sham mice, *n* = 15; T3-treated sham mice, *n* = 15; vehicle-treated TBI mice, *n* = 15; T3-treated TBI mice, *n* = 15.

Mitochondrial metabolism is involved in NSC differentiation^20^. We wanted to understand the implication of mitophagy in T3 control of NSCs’ fates. The results showed that conditioned medium favored NSCs shifting toward a neuronal phenotype, whereas we did not observed this change in NSCs from *Pink1*^−/−^ mice (Fig. 8a–c). Interestingly, we found that conditioned medium from *Pink1*^−/−^ neurons also could not enhance the differentiation of NSCs into mature neurons (Fig. 8d–f). Furthermore, there were no differences in NOR (Fig. 8g) or NSS (Fig. 8h) between the T3 and vehicle treatment groups in *Pink1*^−/−^ mice after TBI. Taken together, these data showed the involvement of mitophagy in T3-promoted neurogenesis and recovery in our model.

## Discussion

Mitochondria are dynamic organelles that play key roles in many cellular functions, including energy production, oxidative stress, metabolism, and cell death^21^. One pathway that neurons employ to prevent excessive ROS production and avoid cell death is effective elimination of dysfunctional or damaged mitochondria via a specialized autophagic process, mitophagy^16^. Impaired mitophagy causes an accumulation of dysfunctional mitochondria, resulting in the neuronal death^12,16^. In this study, our data showed that T3 markedly reduced ROS production and prevented neuronal death by promoting the clearance of damaged mitochondria via *Pink1*-dependent mitophagy. In addition, T3 regulated NSC differentiation toward a neuronal phenotype via crosstalk between mature neurons and NSCs. Taken together, these findings suggested that T3 treatment might provide a therapeutic approach for TBI by preventing cell death and promoting neurogenesis.

Mitophagy plays a key role in mitochondrial quality control with fusion and fission dynamics that eliminate dysfunctional and damaged organelles^16,22^. The mitophagic machinery could eliminate damaged mitochondria that would have otherwise possibly induced apoptosis. Cyt *c* released from damaged mitochondria into the cytosol is an apoptogenic factor that generates death signals in TBI^3,16^. The removal of damaged mitochondria by mitophagy significantly attenuates mitochondria-dependent apoptosis and subsequently reduces neuronal death^16^. Dynamin-related protein 1 (Drp1) is involved in mitochondrial fission and clearance of damaged mitochondria via mitophagy; it is activated and then recruited to mitochondria from the cytosol in response to stress. T3 promotes Drp1 activation and localization to mitochondria^23^. In this study, our data demonstrated that T3 treatment reduced cell death and improved outcomes after TBI. Mitophagic agonists might show potential as new neuroprotective drugs for TBI, but the specific mechanisms involved in promoting the degradation of damaged mitochondria remain to be delineated.

The microenvironment is critical for fate determination and cell differentiation of NSCs^24^. Emerging evidence reveals that TBI markedly augments the proliferation of these cells, but a microenvironment of accumulated toxins, ROS, and reduced oxygen leads to low survival rates for mature neurons^24,25^. In this study, T3 treatment enhanced the clearance of damaged mitochondria via mitophagy and reduced ROS production. Surprisingly, *Pink1* knockout mice demonstrated little recovery after T3 treatment, suggesting the involvement of mitophagy in T3-induced neurogenesis and neuroprotection.

TH has been speculated to be a putative cure that could determine the cell fate, eventual survival, and differentiation of NSCs^6,7^. This notion is inspired by several studies wherein a role for TH has been identified as playing a key role in the modification of mitochondrial metabolism and promotion of differentiation^20^. Recent evidence now suggests that mitochondrial dysfunction is characterized by impaired mitochondrial respiration and increased ROS production, both of which have been shown to impair adult neurogenesis^6,7,24^. In this study, T3 promoted adult neurogenesis via neuron–NSC crosstalk, in which mitophagy was required.

In summary, the present study showed that T3 treatment promoted mitophagy to reduce cell death but also regulated NSC differentiation toward a neuronal phenotype after TBI. Furthermore, we found that T3-induced neurogenesis was via crosstalk between mature neurons and NSCs, in which mitophagy was required. Given that mitophagy is affected in the development of several diseases, further investigation is required to determine how to mediate mitophagy in order to attenuate cell death and improve outcomes in TBI and other mitophagy-related conditions.

## Supplementary information

supplementary figure1

supplementary figure2

## Research involving animals

All animal experiments were approved by the Animal Care and Use Committee and conducted in accordance with the experimental guidelines of the NMU.
