# Supplementary figures and images for: Dual effects of thyroid hormone on neurons and neurogenesis in traumatic brain injury
Source: Cell Death Dis. 2020 Aug 9;11(8):671. doi: 10.1038/s41419-020-02836-9 (PMC7442821; doi:10.1038/s41419-020-02836-9)

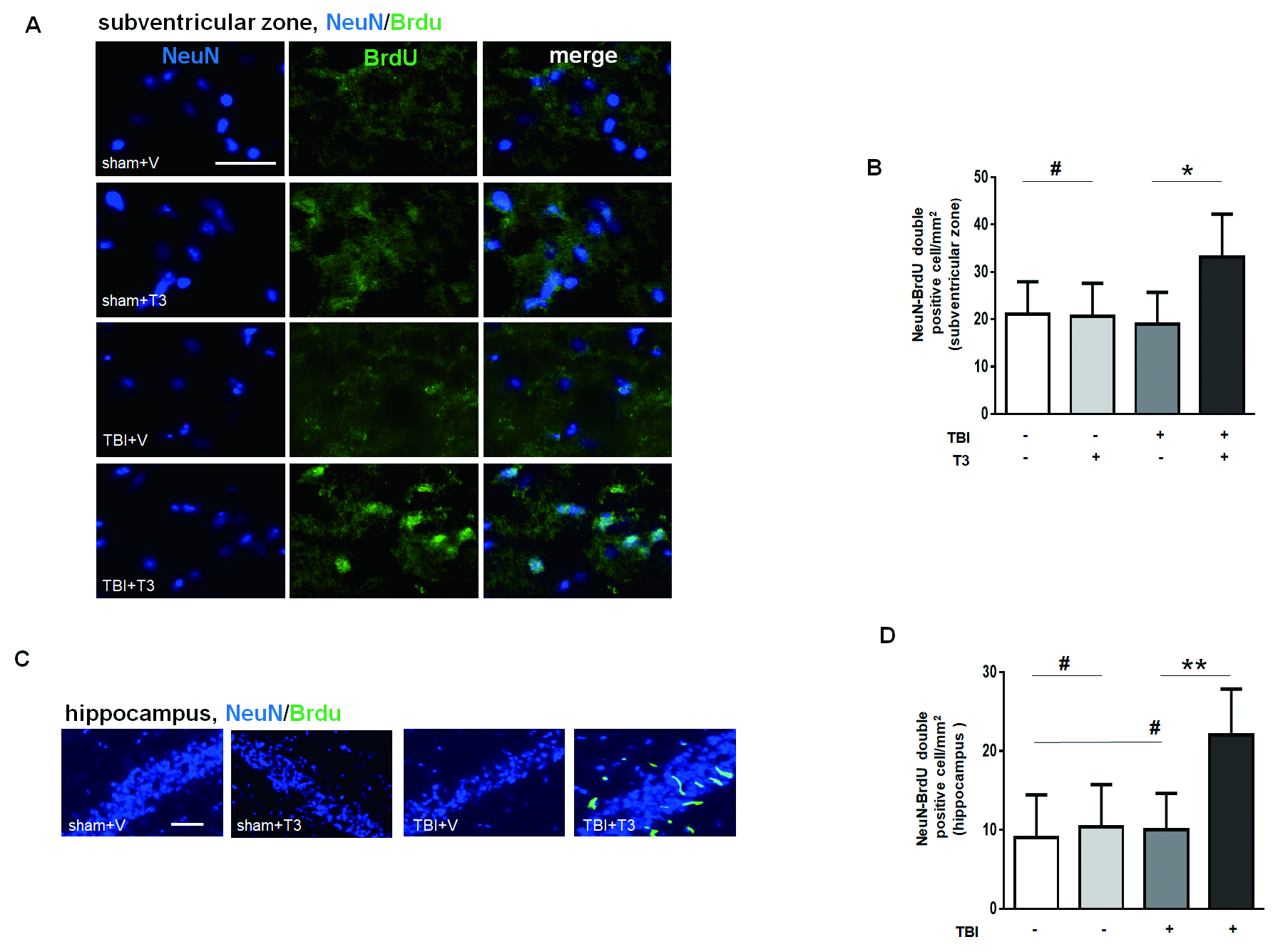

Supplement: Supplementary file 1 — supplementary figure1 [file 41419_2020_2836_MOESM1_ESM.tif]

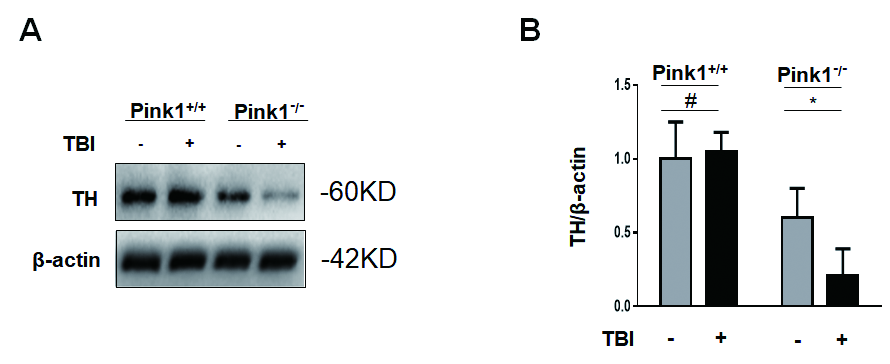

Supplement: Supplementary file 2 — supplementary figure2 [file 41419_2020_2836_MOESM2_ESM.tif]
